# Characterization of pulmonary intimal sarcoma cells isolated from a surgical specimen: *In vitro* and *in vivo* study

**DOI:** 10.1371/journal.pone.0214654

**Published:** 2019-03-29

**Authors:** Takayuki Jujo Sanada, Seiichiro Sakao, Akira Naito, Hatsue Ishibashi-Ueda, Masaki Suga, Hiroki Shoji, Hideki Miwa, Rika Suda, Shunichiro Iwasawa, Yuji Tada, Keiichi Ishida, Nobuhiro Tanabe, Koichiro Tatsumi

**Affiliations:** 1 Department of Respirology (B2), Graduate School of Medicine, Chiba University, Chiba City, Japan; 2 Department of Advanced Medicine in Pulmonary Hypertension, Graduate School of Medicine, Chiba University, Chiba City, Japan; 3 Department of Advancing Research on Treatment Strategies for respiratory disease, Graduate School of Medicine, Chiba University Chiba City, Japan; 4 Department of Pathology, National Cerebral and Cardiovascular Center, Osaka, Japan; 5 Department of Cardiovascular Surgery, Graduate School of Medicine, Chiba University, Chiba City, Japan; University of Texas Health Science Center at San Antonio, UNITED STATES

## Abstract

Pulmonary intimal sarcoma (PIS) constitutes a rare sarcoma originating from the intimal cells of pulmonary arteries. The pathogenesis of PIS remains to be elucidated and specific treatments have not been established; therefore, prognosis is generally poor. The purpose of our study was to isolate and characterize PIS cells from a specimen resected from a patient with PIS. The surgical specimen was minced and incubated, and spindle-shaped and small cells were successfully isolated and designated as PIS-1. PIS-1 cells at passages 8–9 were used for all *in vitro* and *in vivo* experiments. Immunocytochemistry showed that PIS-1 cells were positive for vimentin, murine double minute 2, and CD44 and negative for α-smooth muscle actin, CD31, von Willebrand factor, and desmin. PIS-1 cells exhibited the hallmarks of malignant cells including the potential for autonomous proliferation, anchorage-independent growth, invasion, genetic instability, and tumorigenicity in severe combined immunodeficiency mice. The PIS-1 cells highly expressed tyrosine kinase receptors such as platelet-derived growth factor receptor, and vascular endothelial growth factor receptor 2. Pazopanib, a multi-targeted tyrosine kinase inhibitor, suppressed the proliferation of PIS-1 cells *in vitro* and the growth of tumors formed from xenografted PIS-1 cells. A PIS cell line was thus successfully established. The PIS-1 cells highly expressed tyrosine kinase receptors, which may be a target for treatment of PIS.

## Introduction

Pulmonary intimal sarcoma (PIS) constitutes a subtype of primary sarcoma originating from pulmonary arteries [1]. PIS is recognized as “tumours of uncertain differentiation” according to World Health Organization (WHO) classification [2]. PIS is extremely rare [2,3], although the precise prevalence is unclear. To date, only 300 cases have been documented since the first case report by Mandelstamm in 1923 [4]. The average age at diagnosis is 46 years, and PIS is slightly more prevalent in women (male: female = 1:3) [2]. PIS tumors may originate from unknown mesenchymal cells [5]; however, the detailed: pathogenesis remains unclear. PIS grows within the lumen of pulmonary arteries and eventually occludes those vessels [2]. Common symptoms of primary pulmonary arterial sarcoma include dyspnea, chest pain, edema, cough, and hemoptysis [1,6]. Computed tomography (CT) is characterized by pulmonary artery luminal narrowing or occlusion [1,7]. The clinical characteristics of PIS are similar to those of chronic thromboembolic pulmonary hypertension (CTEPH). Therefore, PIS is extremely difficult to differentiate from CTEPH before surgery [7,8] and even in autopsy [9]. The prognosis of PIS is poor. It was reported that the median survival is 13–18 months [2].

No standard therapy has been established for PIS, although complete surgical resection [1,10] or multimodal therapy [11] might improve the prognosis. Regarding chemotherapy, cytotoxic agents such as adriamycin, and ifosfamide have been empirically used [1,11] as no effective regimen of chemotherapy for PIS has been identified. Furthermore, although proangiogenic proteins may be relevant to tumor growth and serve as potential treatment targets for sarcomas originating from major vessels [12], and several molecularly targeted agents have recently been utilized in various types of sarcoma [13], the appropriate therapeutic molecular target for PIS remains undefined.

We previously successfully isolated and characterized cells from CTEPH surgical specimens [14–16]. Notably, the isolated cells from one patient exhibited malignant potential and formed intravascular tumors within pulmonary arteries in an animal model, which mimicked PIS [16]. In similar methods, spindle-shaped and atypical cells were successfully isolated from surgical specimens and named pulmonary artery intimal sarcoma-1 (PIS-1). The purpose of our study was to characterize these PIS-1 cells *in vitro* and *in vivo*.

## Materials and methods

### Ethical approval and consent to participate

This study was approved by the by the Research Ethics Committee of Chiba University School of Medicine (approval number 353) and Chiba University Instrumental Animal and Use Committee (approval numbers 28–416 and 29–125). Written informed consent was obtained from the patient.

### Cell isolation

The methods of cell isolation were similar to those described in our previous reports [14,16]. Resected specimens were washed with phosphate-buffered saline (PBS) and minced to 3-mm size. The minced tissues were divided into two groups according to the incubation medium. Minced tissues were plated into 6-cm dishes coated with human fibronectin (Corning Inc, Armonk, NY, USA) and incubated with RPMI-1640 medium (Sigma-Aldrich Co, St. Louis, MO, USA) and 10% fetal bovine serum (FBS) (Sigma-Aldrich) at 37°C in a 5% CO_2_ air humidified incubator. In one of these dishes, small spindle-shaped cells were found and named PIS-1. Passage of PIS-1 cells was performed upon reaching 80–90% confluency and Accutase (Thermo Fisher Scientific, Waltham, MA, USA) was used for cell dissociation. In this study, PIS-1 cells at passages 8–9 were used for all *in vitro* and *in vivo* experiments. Additional minced tissues were similarly incubated using endothelial cell growth medium (EGM-2) (Lonza Inc, Allendale, NJ, USA) and 5% FBS, from which endothelial-like (EC-like) cells were obtained. EC-cells were used as the negative control in some experiments.

### Cell lines

To date, there are no cell lines of mesenchymal malignant cells derived from pulmonary arteries available. We used lung adenocarcinoma cells as the positive control in some experiments. The lung adenocarcinoma A549 cell line was purchased from TaKaRa Biomedical (Ohtsu, Shiga, Japan). A549 cells were incubated similarly to PIS-1 cells. The normal mouse fibroblast cells (BALB/3T3) were used as the negative control in the invasion assay. BALB/3T3 cells were adopted from the Department of Biochemistry and Molecular Pharmacology, Graduated School of Medicine, Chiba University. BALB/3T3 cells were incubated in 6-cm dishes coated with human fibronectin (Corning Inc) and with Dulbecco’s modified eagle medium (Thermo Fisher Scientific) and 10% FBS at 37°C in a 5% CO_2_ air humidified incubator.

### Reagents

Antibodies for immunocytochemistry and immunohistochemistry (IHC) performed at Chiba University were as follows: mouse-anti-human vimentin (1:200, M7020, Dako, Carpinteria, CA, USA), mouse anti-α-smooth muscle actin (SMA) (1:1000, A2547, Sigma-Aldrich), rabbit anti-human von Willebrand factor (vWF) (1:1000, A0082, Dako), rabbit anti-human CD31 (1:1000, ab32457, Abcam, Cambridge, UK), mouse anti-human desmin (1:1, IS606, Dako), mouse anti-CD44 (1:100, ab16728, Abcam), mouse anti-murine double minute 2 (MDM2) (1:200, ab16895, Abcam), rabbit anti-platelet-derived growth factor receptor (PDGFR) α (1:100, ab 65258, Abcam), rabbit anti-PDGFR β (1:100, ab62437, Abcam), rabbit anti-vascular endothelial growth factor receptor 2 (VEGFR2) (1:100, ab131441, Abcam), anti-rabbit IgG conjugated with Alexa-488 or Alexa-594 fluorescent dye (both 1:200, Invitrogen, Carlsbad, CA, USA), and anti-mouse-IgG conjugated with Alexa-594 fluorescent dye (1:200, Invitrogen). Antibodies for IHC performed at National Cerebral and Cardiovascular Center were as follows: mouse anti-vimentin (Dako), mouse anti-CD31 (Dako), mouse anti-CD 34 (Dako), mouse anti-human α- SMA (Dako), rabbit anti-human vWF (1:400, Dako), and mouse anti-human desmin (Dako). Antibodies for western blot analysis were as follows: rabbit anti-PDGFR-α (1:500, ab137789, Abcam); rabbit anti-phosphor PDGFR-α (1:500, ab134068, Abcam); rabbit anti-VEGFR2 (1:500, ab39638, Abcam); rabbit anti-phospho VEGFR2 (1:500, ab5473, Abcam); rabbit anti-lamin A+C (1:1000, ab1085595, Abcam); rabbit anti-β-actin antibodies (1:1000, Cell Signaling Technology, Boston, MA, USA); and stabilized goat anti-rabbit IgG (H+L), which was peroxidase conjugated (1:1000, ThermoScientific).

### Soft agar colony formation

Anchorage-independent growth of cell lines was examined using the CytoSelect 96-well Cell Transformation Assay (CBA-130, Cell Biolabs, Inc., San Diego, CA, USA) according to the manufacturer’s provided data sheet. A mixture of 1.2% agar solution, 2× Dulbecco's modified Eagle medium (DMEM), and 20% FBS was placed into each well of a 96-well plate and the plate was incubated at 4°C for 30 minutes. Then, 75 μl of a cell suspension containing 5 × 10^3^ cells, 1.2% agar solution, 2× DMEM, and 20% FBS was placed on the semisolid soft agar layer of each well. The plate was incubated at 37°C under 5% CO_2_ in a humidified incubator for 15 minutes. The culture medium for each cell line was placed in each well and the cultures were incubated at 37°C, under 5% CO_2_ in a humidified incubator for 7 days. After incubation, the soft agar and cell cultures were dissolved using agar solubilization solution and lysis buffer, respectively. The lysates were stained with Cyquant solution and read using a fluorescence plate reader (infinite F200, Tecan Group Ltd. Zürich, Switzerland).

### Immunocytochemistry

Incubated cells were washed using PBS and fixed using 4% paraformaldehyde (Wako Pure Chemical Industries, Osaka, Japan) for 10 minutes. Slides were blocked with 2% FBS, PBS, and 1% Tween 20 for 30 minutes. Next, the slides were incubated with primary antibodies at 4°C overnight and with secondary antibodies at room temperature for 30 minutes. After counterstaining using 2 μg/ml DAPI, the slides were examined under a fluorescence microscope (Eclipse 80i, Nikon Corp. Tokyo, Japan).

### Immunohistochemistry (IHC)

IHC for surgical specimens was performed using the automated immunostainer BOND-III (Leica, Wetzlar, Germany) at the National Cerebral and Cardiovascular Center (Suita, Osaka, Japan). The Bond Polymer Refine Detection kit (DS9800, Novocastra, Leica Biosystems, Nussloch, Germany) was used as a secondary antibody, with incubation with a post-primary antibody for 8 minutes, polymer for 8 minutes, diaminobenzidine for 10 minutes, and hematoxylin for 5 minutes as counterstain.

Immunofluorescence staining methods for tissues resected from mice were performed at Chiba University. Tissues were fixed using 10% formaldehyde (Wako) and embedded with paraffin. After deparaffinizing, antigen retrieval was performed using pH 6.0 citrate buffer (Abcam). Slides were blocked using 2% FBS, PB, and 0.1% Tween 20 for 30 minutes. The slides were incubated with primary antibodies at 4°C overnight and with secondary antibodies at room temperature for 30 minutes. For fluorescence immunostaining, slides were counterstained with 2 μg/ml DAPI followed by examination under a confocal microscope (FV10i, Olympus, Tokyo, Japan).

### Serum starvation

Cells were plated onto 6-cm fibronectin-coated dishes and incubated with serum-free medium, which did not contain FBS or supplementary growth factors, for three weeks. At 3, 7, 14, and 21 days after seeding, the cells were counted.

### Invasion and migration assay

The invasion ability was evaluated using Matrigel invasion chambers (24-well 8-μm) and Biocoat control culture inserts (Corning) according to the provided data sheet. A cell suspension (2.5 × 10^4^ cells) and serum-free medium were placed into the upper wells, and the incubation medium with FBS was added into the lower wells. The plates were incubated at 37°C under 5% CO_2_ in a humidified incubator for 22 hours. After removal of the medium and non-invading cells, the membranes of the upper chambers were Giemsa-stained using Diff-Quick solutions (Sysmex Corporation, Kobe, Japan), followed by air-drying overnight. The membranes removed from the chambers were prepared on microscopic slides. Invaded and migrated cells were counted using light microscopy (Eclipse 55i, Nikon). The invaded or migrated cell number for each chamber was defined as the average for the cells observed in five fields for each slide. The percent invasion was calculated according to the following formula:

$$\%Invasion=\frac{Average\;of\;invading\;cells\;through\;the\;Matrigel\;insert\;membrane\;}{Average\;of\;migrating\;cells\;through\;the\;control\;insert\;membrane}\times 100$$

### Multicolor fluorescence *in situ* hybridization (mFISH)

To analyze the chromosomal karyotype of PIS-1 cells, mFISH was performed by Chromocenter Inc. (Kobe City, Hyogo, Japan). Human mFISH probes were purchased from MetaSystems GmbH (Altlussheim, Germany). The denaturation of metaphase chromosomes and mFISH probes, hybridization, post-hybridization washes, and fluorescence staining were performed using the methods recommended by the manufacturer. Fluorescence images were captured using an AxioImagerZ2 fluorescence microscope (Carl Zeiss GmbH) equipped with a CoolCubeI CCD camera. Color images were processed using the ISIS mFISH software program (MetaSystems).

### PCR array analysis

The RT^2^ Profiler PCR Array (PAHS-161ZA) (SABiosciences, Frederick, MD, USA) was used to evaluate the mRNA expression of tyrosine kinase receptors (TKRs). The RNeasy mini kit (Qiagen, Venlo, The Netherlands) was used for extracting mRNA from incubated cells. The RT2 first strand kit (SABiosciences) was used for reverse-transcription and the synthesis of cDNA. An ABI 7300 system was used for PCR amplification and calculating threshold cycles (Applied Biosystems, Foster City, CA, USA). A549 cells were used as a control and the differences in expression were presented a fold changes. The details of these methods have been described in our previous report [17].

### Western blot analysis

Western blot analysis was performed based on a modified method described previously[17]. For extracting whole protein of cultured cells, those cells were homogenized using lysis buffer (20 mM Tris-HCl, pH 8.0, 1 mM EDTA, 1 mM NaN_3_, 1 mM DTT, 150 mM NaCl, 0.5% Triton-X, phosphatase inhibitor cocktail (SIGMA P5726)). Extraction of nuclear protein was performed using Minute Cytoplasmic and Nuclear Extraction Kit (Invent Biotechnologies, Inc, Plymouth, MN, US) according to the datasheet. Protein samples were separated on and transferred to nitrocellulose membranes. Membranes were blocked with 5% bovine serum albumin (BSA) or dry fat milk in tris-buffered saline (TBS) containing 0.5% Tween 20 (TBS-T) for one hour at 4°C. Next, the membranes were incubated with primary antibodies diluted to TBS-T containing 1% BSA or dry fat milk for 18 hours at 4°C. The membranes were incubated with secondary antibodies diluted to TBS-T containing 1% BSA or dry fat milk for one hour at room temperature. Chemiluminescence was detected using a LAS-1000 (Fuji Film, Tokyo, Japan).

### Subcutaneous and intravenous tumorigenicity

Male severe combined immunodeficiency (SCID) mice (CB-17/Icr-scid/scid-Jcl, aged five weeks) were purchased from CLEA Japan, Inc. (Tokyo, Japan). All mice were housed in a room with a 12 hour light/dark cycle (6am to 6pm) at 24°C in the animal experimentation facility of Chiba University. Mice were kept in sterilized plastic cages (size 18.2×26.0×12.8cm), and had free access to food and distilled water. Cages, food and water were replaced once a week. Anesthetic agents containing medetomidine (0.3mg/kg), midazolam (4mg/kg), and butorphanol tartrate (5mg/kg) were injected intraperitoneally to mice before all procedures. PIS-1 cells (2 × 10^6^) were injected subcutaneously on the back or intravenously into SCID mice. Injected mice were sacrificed by blood removal, and the organs were resected at the appropriate time. When animals lost more than 20% of body weight per week, they were euthanized by blood removal for humane reasons.

### Labeling cells with PKH-26

To label and trace the injected cells in mice, PKH-26 (Sigma-Aldrich) was used. Details of the labeling methods were described in our previous report [16]. Labeled cells were injected according to methods described above. Resected specimens were fixed using 4% paraformaldehyde (Wako) for 72 hours, embedded in Tissue-Tek O.C.T compound (Sakura Finetek Japan Co. Ltd., Tokyo, Japan), and frozen at −80°C. Specimens were sliced into 3-μm sections. Slides were washed using PBS and counterstained with 2 μg/ml DAPI. PKH-positive cells were detected using a fluorescence microscope (Eclipse 80i).

### Cell culture with a tyrosine kinase inhibitor

To evaluate the effects of tyrosine kinase inhibitors on PIS-1 cells, pazopanib, a multi-targeted tyrosine kinase inhibitor (TKI), was used. Pazopanib was purchased from SYNkinase (Manning Bridge, Australia), stored at −20°C, and prepared just before use. Pazopanib was diluted with dimethyl sulfoxide (DMSO) and a solution of 10 mM pazopanib was prepared. The solution was diluted with incubation medium containing 10% FBS for incubation. Then, 1 × 10^5^ PIS-1 cells were incubated with 0.1, 1.0, or 10 μM pazopanib according to previous reports [18,19]. The control group was incubated with incubation medium containing 10% FBS and 0.1% DMSO, where the concentration of DMSO of the control group was similar to that of the 10 μM pazopanib group. At days 3, 7, 10, and 14, the cell numbers were counted.

### Treatment of xenografted mice with a tyrosine kinase inhibitor

To evaluate the effects of a TKI in xenografted mice, pazopanib was orally administered to mice according to previous reports [20,21]. Pazopanib was suspended using 0.5% hydroxypropyl methylcellulose (Sigma Aldrich) with 1% Tween 80. Mice that were subcutaneously injected with 2 × 10^6^ PIS-1 cells on their right back were assigned to two groups at random: 1) a group orally treated with pazopanib (40 mg/kg) twice daily; (pazopanib group); 2) a group treated with vehicle without pazopanib (control group). Subcutaneous tumor size was measured using calipers, and tumor size was calculated according to the following formula, as described in a previous report [20]:

$$Tumor\;size\;({mm}^{3})=\frac{a\times b^{2}}{2}$$

where “a” and “b” are the longest and shortest diameter of the subcutaneous tumor, respectively. After 14-day treatments, mice were sacrificed and the weights of resected tumors were measured.

### Statistical analysis

PCR array analysis was performed using the web-based software, RT2 Profiler PCR Array Data Analysis, ver. 3.5 (http://pcrdataanalysis.sabiosciences.com/pcr/arrayanalysis.php). The other data were analyzed using the statistical software, EZR on R commander (ver. 1.35, Saitama Medical Center, Jichi Medical University, Saitama, Japan) [22]. Comparisons between two groups or among three groups were analyzed using Student’s t-test and one-way ANOVA, respectively. *p* < 0.05 was considered to be significant. Continuous variables were described as the mean ± standard deviation unless otherwise stated.

## Results

### Pathological evaluation of surgical specimens

**Fig 1:**
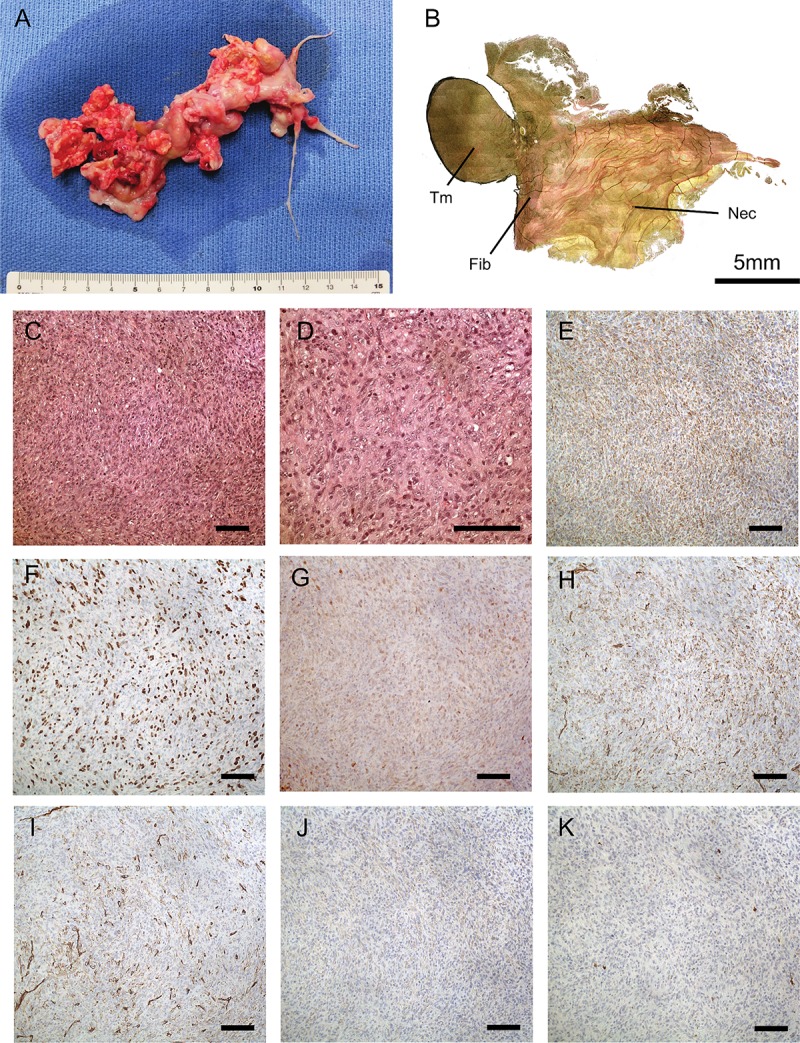
Primary tumor and pathological slides. (A). Photo-macrography of the resected specimen of PIS. B: Composite photograph of the specimen derived from the left main pulmonary artery stained by elastic-van Gieson staining. The specimen contained different tumor, fibrotic, and necrotic components. (C) Low magnification photomicrographs (×200) with hematoxylin-eosin (HE) staining; (D) High-magnification photomicrographs with HE staining (×400). E-J were photomicrographs with immunohistochemistry. (E) Vimentin, (F) Murine double minute 2 (MDM2), (G) Cyclin-dependent kinase 4 (CDK4), (H) CD31, (I) CD34, (J) α-smooth muscle actin. (K) S-100 protein. Tm: tumor component; Fib: fibrotic component; Nec: necrotic component. Scale bar shows 100 μm unless otherwise stated.

Pathological evaluation revealed that the specimens consisted of several components: necrotic, fibrotic, and tumorous (Fig 1A). The tumorous components were found in the specimen derived from the left main pulmonary artery (Fig 1B). Fig 1C and 1D show high cellularity of pyknotic atypical cells in low- and high-magnification photomicrographs stained with hematoxylin-eosin (HE). Immunohistochemistry showed that the tumor cells were diffusely positive for vimentin (Fig 1E), MDM2 (Fig 1F), and CDK4 (Fig 1G), partially positive for CD31 (Fig 1H) and CD34 (Fig 1I), and weakly positive for α-SMA (Fig 1J). Tumor cells were negative for S-100 protein (Fig 1K). Most (60–70%) tumor cells were positive for the proliferation marker Ki-67. Based on these findings, an expert pathologist (Dr. I.-U.) diagnosed the tumor as undifferentiated PIS.

### Characteristics of isolated cells

**Fig 2:**
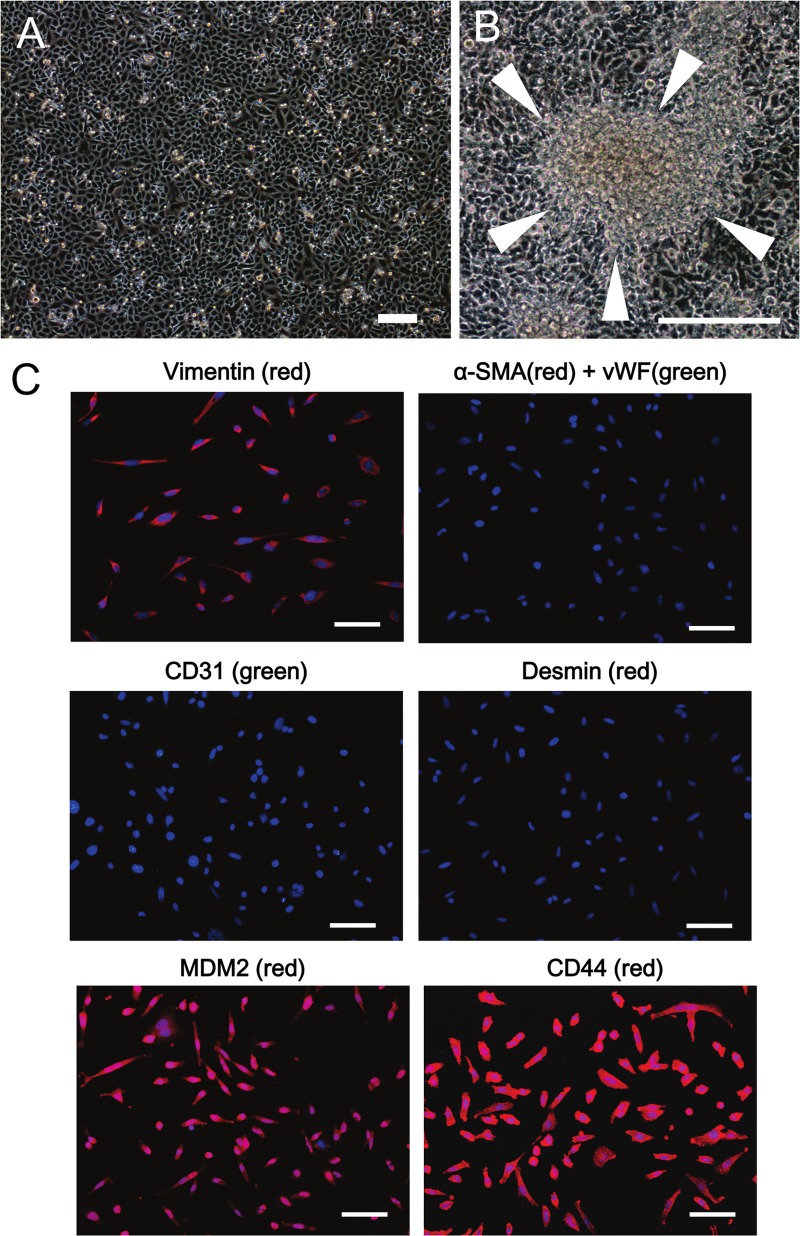
Characteristics of PIS-1 cells *in vitro*. (A) PIS-1 cells present as small and spindle-shaped cells microscopically. Scale bar, 200 μm. (B) Transformed foci of PIS-1. Scale bar, 300 μm. (C) Immunocytochemistry shows that PIS-1 cells were diffusely positive for vimentin, murine double minute 2 (MDM2), and CD44, and negative for α-smooth muscle actin (SMA), von Willebrand factor (vWF), CD31, and desmin. Scale bars, 50 μm. Nuclei stained with DAPI are represented by blue.

Spindle-shaped small cells were successfully isolated from surgical specimens incubated with RPMI-1640 and designated as PIS-1 (Fig 2A). The PIS-1 cells appeared to form transformed foci (Fig 2B). Immunocytochemistry revealed that PIS-1 cells were positive for vimentin, MDM2, and CD44, but negative for α-SMA, vWF, CD31, and desmin (Fig 2C). Immunocytochemistry also demonstrated that 40–50% of PIS-1 cells were positive for Ki-67. Additional cells isolated from specimens that were incubated with EGM-2 were designated as EC-like cells (S1A Fig). EC-like cells were round and did not form any layered structures such as transformed foci. Immunocytochemistry revealed that the EC cells were positive for vimentin (S1B Fig), vWF (S1C Fig), and CD31 (S1D Fig), but negative for desmin (S1E Fig).

### Genetic instability

**Fig 3:**
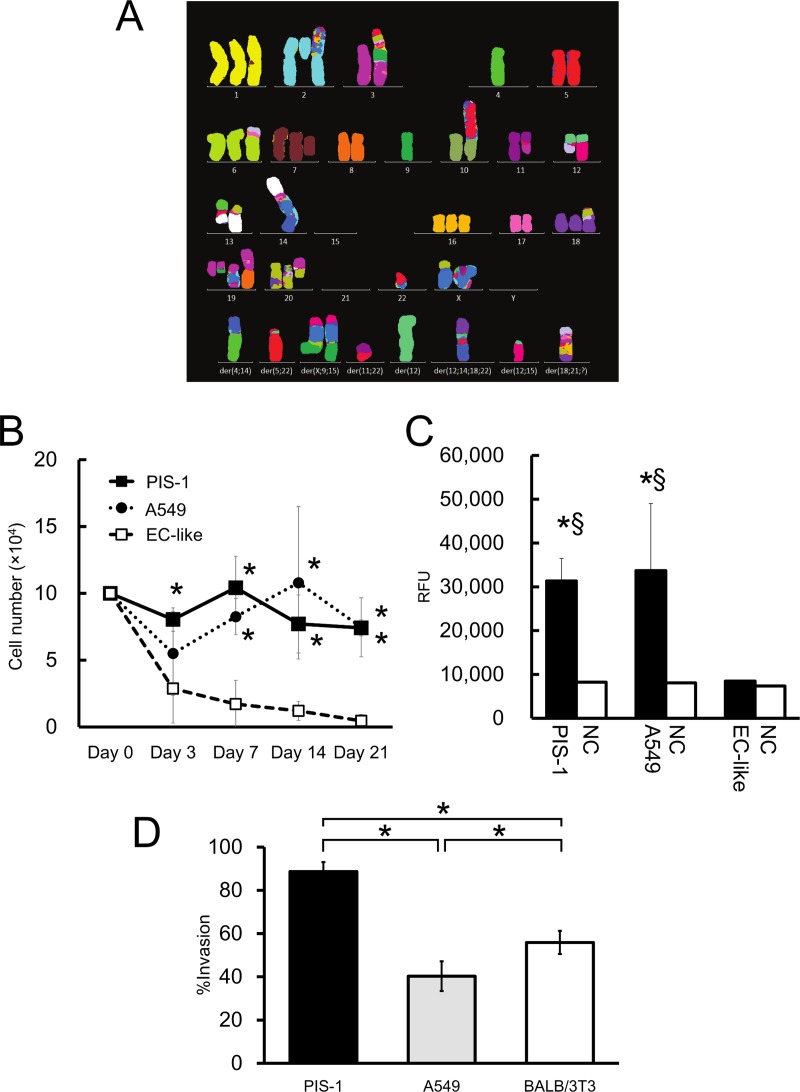
Malignant potential of PIS cells. (A) Representative multicolor fluorescence *in situ* hybridization (mFISH) karyogram from PIS-1 cells. (B) Starvation assay. Three cell groups (PIS-1, A549, EC-like) were incubated with each serum-free medium. *p < 0.05 vs. EC-like cells. (C) Colony formation assay. Cell suspensions containing 5 × 10^3^ PIS-1, A549, or EC-like cells were seeded on the semisolid soft agar layer. After seven days of incubation, the formed colonies and soft agar were dissolved and stained with fluorescent dye followed by reading with a fluorescence plate reader. RFU: relative fluorescence units, NC: negative control, *p < 0.05, vs EC like cells; *§p* < 0.05, vs negative control of each group. (D) Invasion assay. To evaluate the invasion and migration potential of PIS-1 cells, Matrigel invasion chambers (24-well) and Biocoat control culture inserts were used. BALB/3T cells were used for negative control according to the datasheet. Cell suspension (2.5 × 10^4^) and serum-free medium were placed into the upper wells, and the incubation medium with FBS was added into lower wells. After 22 hours of incubation, the cells that migrated or invaded through membranes were counted microscopically. The percent invasion was defined as the ratio of invaded to migrated cells. Error bars show standard deviation. *: *p* < 0.05.

mFISH analysis showed the karyotype abnormalities of PIS-1 cells (Fig 3A). The most common chromosome number within the 20 analyzed cells was 56 (range 51–59). The karyogram showed extremely complicated abnormalities of chromosomes such as duplications, deletions, and derivatives. Therefore, the definite karyotype of PIS-1 was not identified. Detailed mFISH data are described in S1 Table.

### Serum-independent growth

Autonomous proliferation was evaluated by incubation with serum-free medium. PIS-1 cells exhibited the potential for serum-independent growth. Both PIS-1 and A549 (lung cancer) cell lines had proliferated more than EC-like cells at days 7, 14, and 21 after incubation (Fig 3B).

### Anchorage-independent proliferation

To evaluate the capacity for anchorage-independent proliferation, a colony formation assay was performed. The colony formation assay showed that PIS-1 cells exhibited the potential for anchorage-independent growth. PIS-1 cells proliferated and formed colonies in soft-agar as well as A549 cells, whereas no colonies were formed by EC-like cells (Fig 3C).

### Invasion and migration activity

The invasion assay revealed the invasive activity of the PIS-1 cells. The percent invasions of PIS-1, A549, and BALB/3T3 cells were 88.7 ± 4.4%, 40.3 ± 6.9%, and 55.9 ± 5.4%, respectively. The value of PIS-1 was significantly higher than those values of A549 and BALB/3T3 cells (vs A549: *p* = 0.0001, and vs BALB/3T3: p = 0.001). (Fig 3D).

### Tumorigenicity in mice

**Fig 4:**
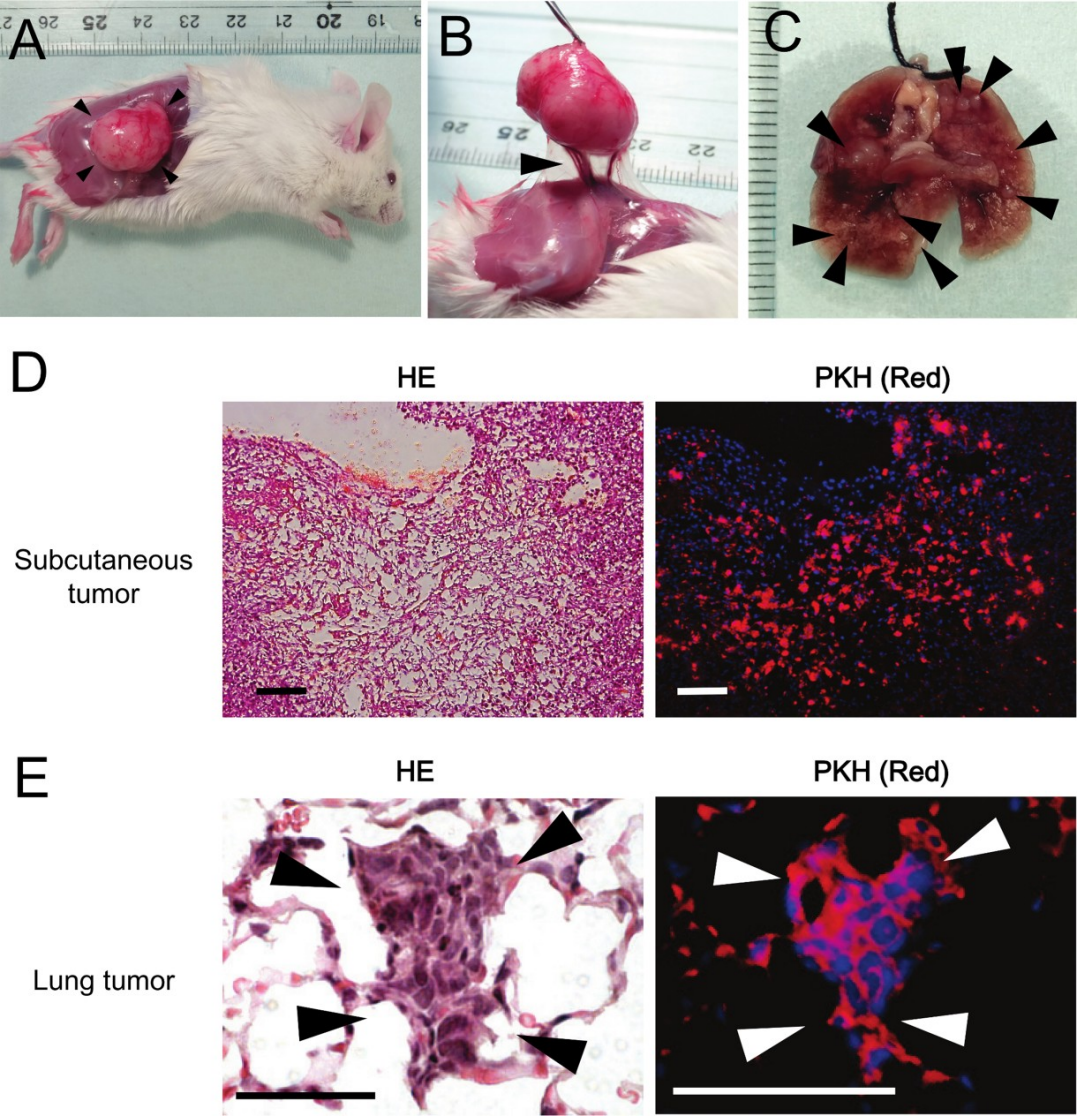
Tumorigenicity of PIS-1 cells. PIS-1 cells (2 × 10^6^) were injected subcutaneously or intravenously into a male C.B-17/lcr-scid/scidJcl (SCID) mouse. (A) Subcutaneous enlarged tumor 28 days after subcutaneous injection (arrow head). Skin around the tumor was removed. (B) Neo tumor vessels were connected with the subcutaneous tumor and the back of the mouse (arrow head). (C) Multiple lung tumors 49 days after intravenous injection (arrow heads). (D) PIS-1 cells labeled by PKH-26 were present at the central area of a subcutaneous tumor. Scale bars, 100 μm. (E) Lung tumors were composed of cells positive for PKH-26. Scale bars, 50 μm. Nuclei stained with DAPI are represented by blue.

To evaluate the tumorigenicity of PIS-1 cells, the cells were injected subcutaneously or intravenously to SCID mice. Tumors formed in all injected mice. Fig 4A shows s subcutaneous tumor formed from PIS-1 cells 28 days after subcutaneous injection. The tumor was round and opalescent, with neovascular vessels connecting the tumor and the back of the mouse (Fig 4B). Invasion to the surrounding tissues was not observed in any mice. Fig 4C shows multiple lung tumors formed from PIS-1 cells after intravenous injection. PIS-1 cells labeled by PKH-26 were detected within the subcutaneous and lung tumors (Fig 4D). No metastatic lesion was detected in any mice regardless of subcutaneous or intravenous injection.

### High expression of tyrosine kinase receptors (TKRs)

**Fig 5:**
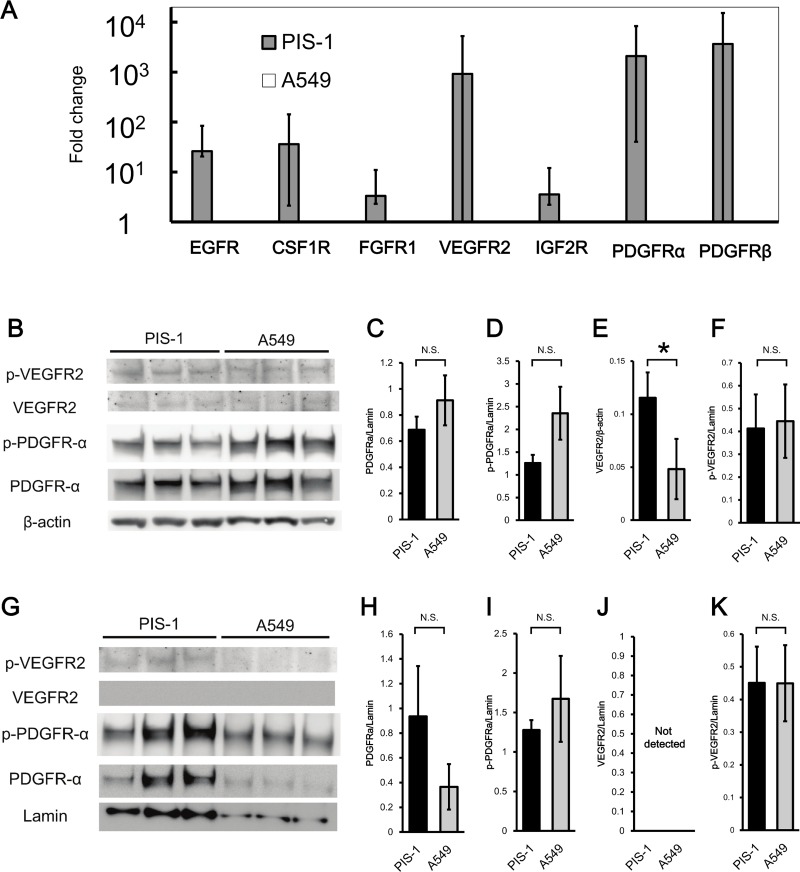
PCR array and western blot analysis for tyrosine kinase receptors. (A) PCR array analysis showing expression of tyrosine kinase receptors in PIS-1 cells in comparison to A549 cells. Vertical axis is in log scale. (B) Western blot analysis for whole protein of PIS-1 and A549 cells. The signal strength of platelet-derived growth factor receptor (PDGFR)-α (C), phospho-PDGFR-α (D), vascular endothelial growth factor receptor 2 (VEGFR2) (E), and phospho-VEGFR2 (F) were expressed as a ratio to β-actin. *:p<0.05 vs A549 cells. (G) Western blot analysis for nuclear protein of PIS-1 and A549. The signal strength of PDGFR-α (H), phospho-PDGFR-α (I), VEGFR2 (J), and phospho-VEGFR2 (K) were expressed as a ratio to lamin. Error bars show standard deviation.*:p < 0.05 vs A549 cells, N.S.: not significant.

**Fig 6:**
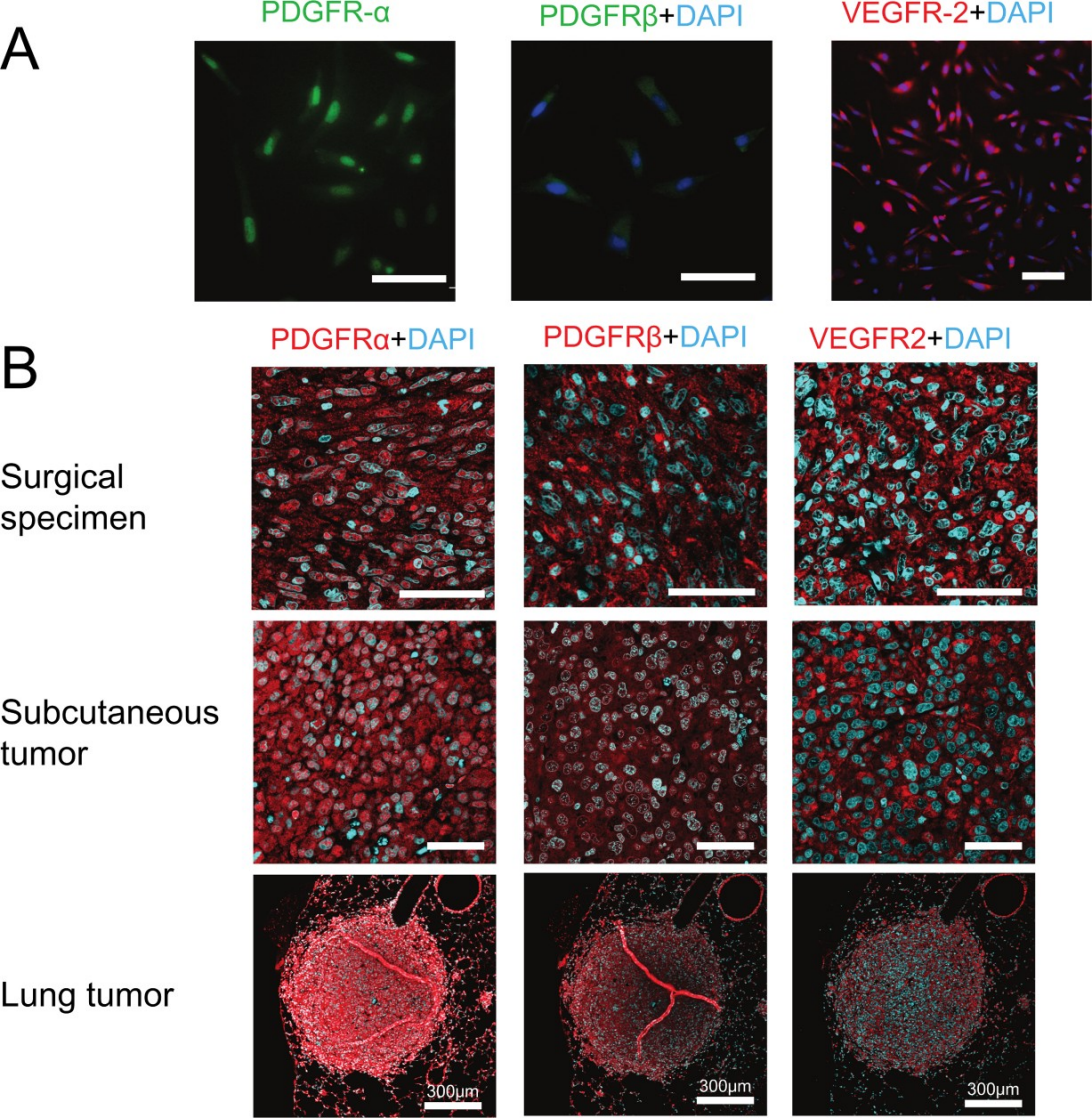
Immunocytochemistry and immunohistochemistry for tyrosine kinase receptors in PIS-1 cells. (A) Immunocytochemistry shows that PIS-1 cells were positive for platelet-derived growth factor receptor (PDGFR)-α, PDGFR-β, and vascular endothelial growth factor receptor (VEGFR)-2. (B) Immunohistochemistry shows that the tumor components of the surgical specimen, and subcutaneous and pulmonary tumors formed from PIS-1 cells and were diffusely positive for PDGFR-α, PDGFR-β, and VEGFR-2. All scale bars, 50 μm unless otherwise stated.

To explore other characteristics and treatment targets of PIS-1 cells, a PCR array was performed. PCR array analysis revealed high expression of mRNA associated with TKRs (Fig 5A and Table 1). Western blot analysis for whole protein revealed that the expression of VEGFR2 in PIS-1 cells was significantly higher than that of A549 (p = 0.035) (Fig 5B–5F). Western blot analysis for nuclear protein showed that the expression of PDGFR-α in PIS-1 cells was higher than that of A549, although the difference was not significant (p = 0.09). Immunocytochemistry showed that PIS-1 cells were positive for PDGFR-α and VEGFR-2, and weakly positive for PDGBR-β (Fig 6A). IHC showed that tumor cells of the surgical specimen and subcutaneous and lung tumors of PIS-1 cells were positive for PDGFR-α, PDGBR-β, and VEGFR-2 (Fig 6B).

**Table 1:** Expression of tyrosine kinase receptors of PIS-1 on PCR array.

Description	Gene symbol	Public ID	Fold change (95%CI)	*p*-value
Epidermal growth factor receptor	*EGFR*	NM_007912	26.1008 (20.37–31.83)	0.000006
Colony stimulating factor 1 receptor	*CSF1R*	NM_001037859	36.0856 (2.12–70.05)	0.013473
Fibroblast growth factor receptor 1	*FGFR1*	NM_010206	3.3167 (2.31–4.32)	0.001177
Fibroblast growth factor receptor 2	*FGFR2*	NM_010207	0.0369 (0.03–0.05)	0.001693
Fibroblast growth factor receptor 3	*FGFR3*	NM_008010	0.5912 (0.27–0.91)	0.156391
Fibroblast growth factor receptor 4	*FGFR4*	NM_008011	0.0048 (0–0.01)	0.010772
Insulin-like growth factor I receptor	*IGF1R*	NM_010513	1.7656 (1.38–2.15)	0.003032
Insulin-like growth factor 2 receptor	*IGF2R*	NM_010515	3.5537 (2.2–4.91)	0.010932
Vascular endothelial growth factor receptor 1	*VEFGR1*	NM_010228	4.1943 (0.00001–9.04)	0.128946
Vascular endothelial growth factor receptor 2	*VEFGR2*	NM_010612	920.5719 (0.00001–3416.92)	0
Vascular endothelial growth factor receptor 3	*VEFGR3*	NM_008029	0.5907 (0.0001–1.38	0.316708
Platelet derived growth factor receptor, alpha polypeptide	*PDGFRA*	NM_011058	2097.7398 (40.17–4155.31)	0.000006
Platelet derived growth factor receptor, beta polypeptide	*PDGFRB*	NM_008809	3668.7003 (0.001–7934.79)	0.003171

### Effect of pazopanib on PIS-1 cells

**Fig 7:**
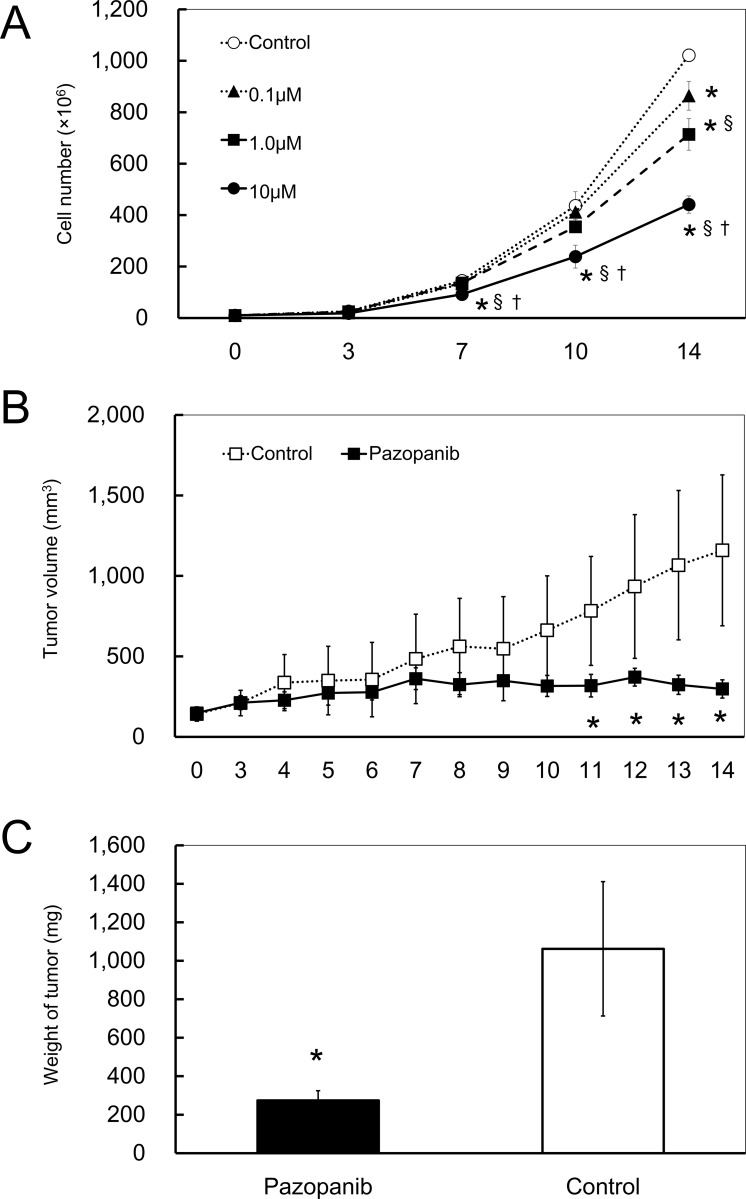
Suppressive effect of pazopanib on the proliferation of PIS-1 cells. (A) Effect of pazopanib, which is a multikinase inhibitor, on the proliferation of PIS-1 cells 14 days after treatment. *p < 0.05, vs control; §*p* < 0.05, vs 0.1 μM; †*p* < 0.05, vs 1.0 μM group. (B) Tumor volumes of mice with 14-day administration of pazopanib compared to those of the control. *p < 0.05, vs control of each day. (C) The mean weights of subcutaneous tumors resected from mice of the pazopanib group compared to those of the control group. Error bars show standard deviation. *p < 0.05, vs control.

To evaluate whether TKRs may constitute treatment targets for PIS, pazopanib, an approved TKI for soft tissue sarcoma and renal cell carcinoma [12], was used for treatment for PIS-1 cells and the xenograft mice. Pazopanib significantly suppressed the proliferation of PIS cells *in vitro* (Fig 7A) and also suppressed the growth of subcutaneous tumors (Fig 7B). The weights of the resected tumors of mice in the pazopanib group were significantly lower than those of tumors from mice in the control group (Fig 7C).

## Discussion

In the current study, PIS cells were successfully isolated from a surgical specimen of PIS and retained the tumor’s malignant potential. The results demonstrated that the PIS-1 cell line highly expressed some TKRs and that PIS-1 cell proliferation could be suppressed by multi-target (PDGFR and VEGFR) TKI agents both *in vitro* and *in vivo*.

PIS-1 cells derived from a surgical specimen and retained the tumor’s malignant potential. Hanahan and Weinberg suggested that the hallmarks of malignant cells comprised a resistance to cell death, cell immortalization, sustained proliferation, insensitivity to growth suppressors, and potential for angiogenesis and invasion [23]. In the current study, the PIS-1 cells exhibited resistance against cell death (Fig 3B), lacked contact inhibition (Fig 2B), and demonstrated anchorage independent proliferation (Fig 3C), invasion capacity (Fig 3D), angiogenesis (Fig 4B), and tumorigenicity (Fig 4A and 4C), indicating that the PIS-1 cells had some hallmarks of malignant cells. We previously reported that the sarcoma-like cells derived from chronic thrombi of CTEPH formed tumors mainly within pulmonary arteries after intravenous injection [16,17]. PIS-1 formed intrapulmonary lesions within capillaries. The reason for this difference was unclear in the current study. It was suggested that isolated PIS cells completely retained the characteristics of malignant cells. Hanahan and Weinberg also described that such a hallmark of malignant cells may be backed by genetic instability [23]. In the present study, it was obvious that PIS-1 cells exhibited numerous chromosomal abnormalities (Fig 3A), suggesting that the PIS-1 cells might act as malignant cells.

The characteristics of histological and cytological analyses in the current study were consistent with previous reports of PIS. It was described that the characteristics of histopathology of PIS is a poorly differentiated mesenchymal tumor, and the tumors are characterized with atypical spindle-shaped cells and necrosis [2]. Several immunohistological markers for PIS have been reported, although specific cell markers have not been identified to date. Vimentin is frequently positive in intimal sarcoma, whereas vascular markers such as CD31, CD34, vWF, and smooth muscle cell markers such as desmin are usually negative [2,24,25]. WHO described that MDM2 is positive in approximately 70% of cases with PIS [2]. Recent studies also reported that PDGFRα and CD44 were positive in intimal sarcoma [25,26].

Our PIS-1 cells may constitute the first established cell line of PIS. Dewaele et al. performed *ex vivo* experiments using primary cells of PIS derived from surgical specimens; however, the primary cells were incubated for only 3 days [26]. To our knowledge, no established cell line of PIS has been generated before the current study. Prognosis has been poor especially in patients with incomplete PIS resection, although complete PIS resection could improve the prognosis [10]. It was suggested that chemotherapy is required, particularly for PIS patients with incomplete resection and inoperable patients. PIS is a rare disease [2]; therefore large clinical trials for chemotherapy of PIS have not been performed. The cell line and xenograft model of PIS-1 cells could contribute reproducible experiments for exploring chemotherapeutic agents and hopefully lead to a breakthrough in the future treatment of PIS-1.

In the present study, PIS-1 cells highly expressed several TKRs, which might serve as targets for chemotherapy. Several studies have described the possible beneficial effects of TKIs including pazopanib for PIS. Funatsu et al. indicated that pazopanib led to a regression of PIS in a case report [27]. Kollár, et al. reported that treatment with pazopanib yielded a partial response in two patients with advanced PIS [12]. In the current study, pazopanib significantly suppressed the proliferation of incubated PIS-1 cells as well as the growth of xenografted tumors. Taken together, the data suggests the effectiveness of pazopanib for PIS. Pazopanib might act on PDGFR and /or VEGFR2, although the precise mechanisms are unclear. PDGFRα was highly amplified in surgical specimens of PIS as determined by FISH analysis in previous studies [26,28]. In the current study, PCR array showed that PIS-1 cells could highly express some TKRs, including PDGFRα. In addition, Dewaele, et al. described that the inhibition of PDGFRα and the related signaling pathways of PIS might constitute a therapeutic target for PIS [26]. In the current study, western blot analysis showed the high expression of VEGFR2 proteins. To our knowledge, the relationship between VEGFR2 and PIS has not been reported, therefore the role of VEGFR2 was unclear. It appears that some TKIs including pazopanib may therefore be effective for PIS and might serve as a treatment option. Clinical trials of TKIs for PIS are needed to verify this conclusion.

There were some limitations in the current study. First, the features of PIS-1 as malignant cells were not comprehensively clarified. Second, the precise mechanisms how pazopanib worked for PIS-1 cells was not investigated in this study. Despite these limitations, we believe that the established cell line and xenograft model may meaningfully contribute to the search for future treatment targets in PIS.

In conclusion, PIS-1 cells to represent the first established cell line of PIS. PIS-1 cells highly expressed some types of TKRs, which may constitute targets for treatment of PIS.

## Supporting information

S1 Fig Characteristics of endothelial-like cells. (A) Endothelial-like cells presented microscopically as small, round cells. Immunocytochemistry shows that endothelial-like cells were positive for vimentin (B), vWF (C), and CD31 (D) and negative for α-smooth muscle actin (SMA) (C) and desmin (E). Scale bar shows 100 μm unless otherwise stated.
(PDF)

S1 Table Details of chromosomal analysis by multicolor fluorescence in situ hybridization. (XLSX)
