# Supplementary figures and images for: Characterization of pulmonary intimal sarcoma cells isolated from a surgical specimen: In vitro and in vivo study
Source: PLoS One. 2019 Mar 29;14(3):e0214654. doi: 10.1371/journal.pone.0214654 (PMC6440640; doi:10.1371/journal.pone.0214654)

A

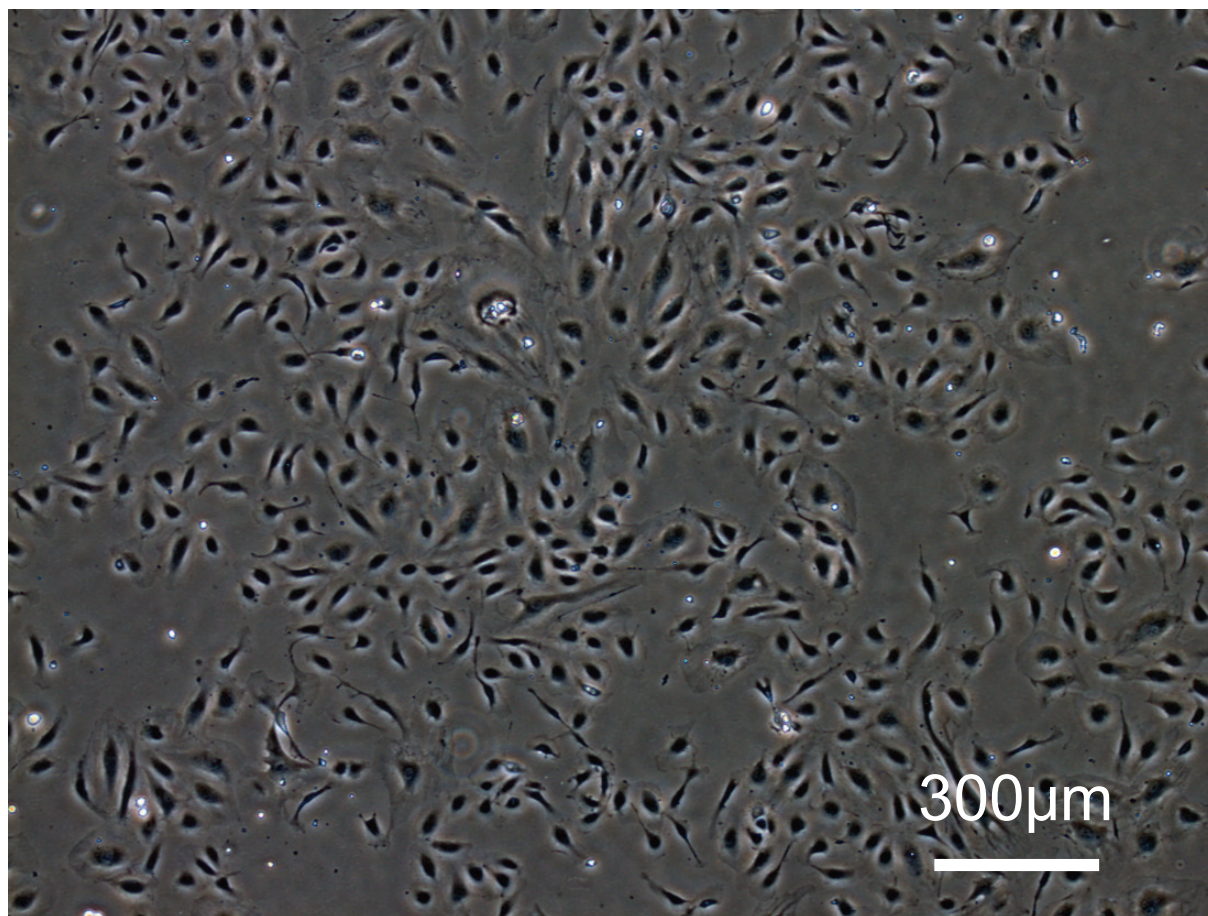

B

Vimentin (red)

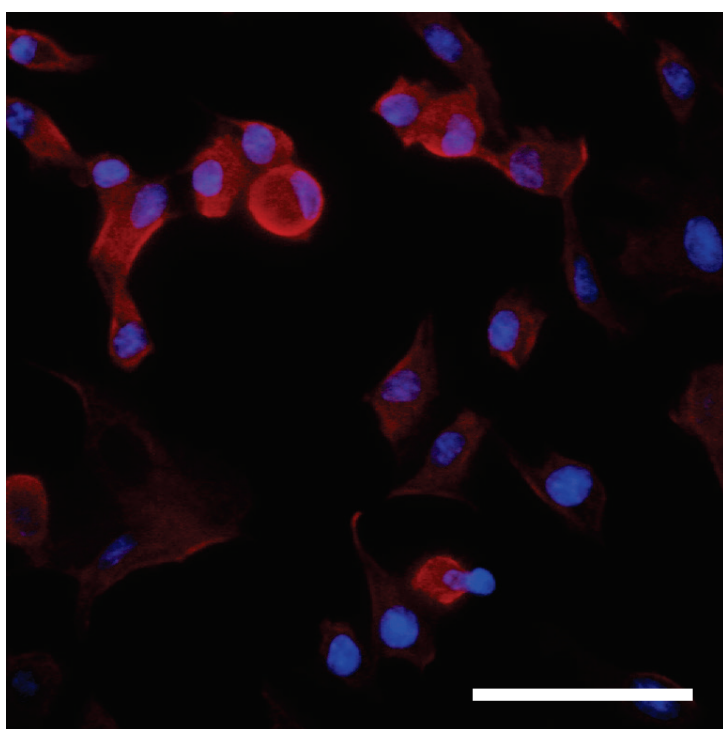

C

Factor VIII (green) +  $\alpha$ -SMA (red)

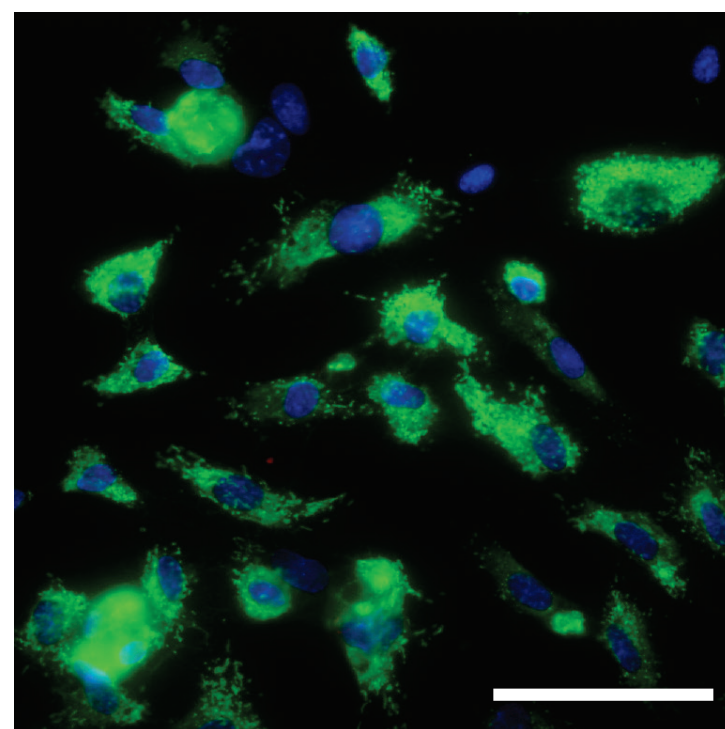

D

CD31 (green)

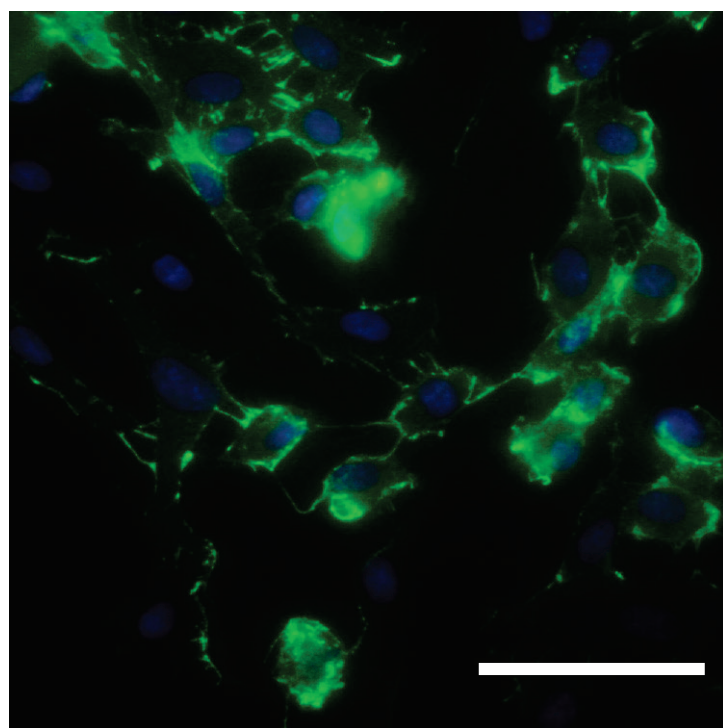

E

Desmin (red)

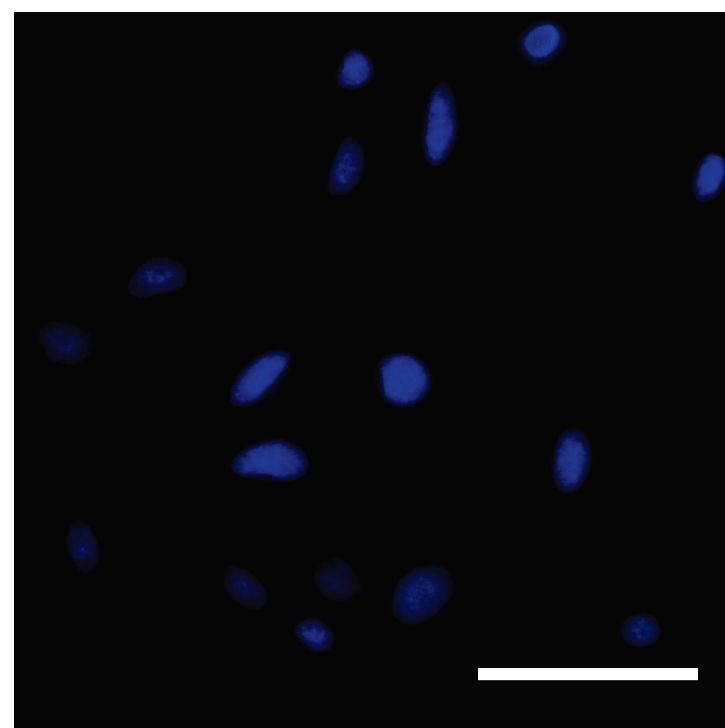

Supplement: S1 Fig — (A) Endothelial-like cells presented microscopically as small, round cells. Immunocytochemistry shows that endothelial-like cells were positive for vimentin (B), vWF (C), and CD31 (D) and negative for α-smooth muscle actin (SMA) (C) and desmin (E). Scale bar shows 100 μm unless otherwise stated. (PDF) [file pone.0214654.s001.pdf]
